# Functional analysis of *TCF7L2* genetic variants associated with type 2 diabetes

**DOI:** 10.1016/j.numecd.2011.12.012

**Published:** 2013-06

**Authors:** D.X. Pang, A.J.P. Smith, S.E. Humphries

**Affiliations:** BHF Centre for Cardiovascular Genetics, Department of Medicine, University College London, 5 University Street, WC1E 6JF London, United Kingdom

**Keywords:** TCF7L2, Diabetes, Polymorphism, Gene expression, Splicing, EMSA, Electrophoretic mobility shift assay, FAIRE, Formaldehyde-assisted isolation of regulatory elements, GWAS, Genome-wide association study, MC-EMSA, Multiplexed competitor-electrophoretic mobility shift assay, PBMC, Peripheral blood mononuclear cell, SIE, cis-inducible element, SNP, Single nucleotide polymorphism, T2D, Type 2 diabetes mellitus

## Abstract

**Background and aims:**

Common non-coding variations within the *TCF7L2* locus have a strong influence on type 2 diabetes (T2D) susceptibility through uncharacterised mechanisms. An islet-specific functional polymorphism has been identified, although this does not explain the association between genotype and gene expression in other cell types. This study sought to identify these other functional *TCF7L2* variants.

**Methods and results:**

Alternative splicing and gene expression from *TCF7L2* was examined from peripheral blood mononuclear cells from 100 healthy Caucasians using two T2D-associated SNPs, rs7903146 and rs12255372. Electrophoretic mobility shift assays and luciferase reporter assays were performed with these SNPs and those in strong LD to determine potential SNP functionality.

Individuals homozygous for rs7903146 and rs12255372 T2D risk alleles (TT/TT) expressed 2.6-fold greater levels of *TCF7L2* mRNA compared to individuals homozygous for the non-risk alleles (CC/GG, *p* = 0.006), although differentially spliced *TCF7L2* transcripts did not differ by T2D risk-associated genotype. From SNPs identified to be in strong LD with the T2D-associated SNPs, rs7903146 and rs12255372, five (rs4132670, rs4506565, rs7903146, rs7901695, rs17747324) demonstrated allele-specific binding in electrophoretic mobility shift assays (EMSA). In luciferase reporter assays, rs4132670 exhibited 1.3-fold higher levels of enhancer activity in the Huh7 cell line (*p* = 3.8 × 10^−5^) and 2-fold higher levels in a WiDr colon carcinoma cell line (*p* = 0.008).

**Conclusions:**

These results suggest that rs4132670, located in a region of chromatin accessibility, is a non-tissue-specific candidate functional SNP that has the potential to play a role in *TCF7L2* gene expression and T2D risk.

## Introduction

The risk of developing T2D is increased by environmental factors such as obesity, sedentary lifestyle and nutrition [1], however, there is a strong genetic component to disease [2]. A genome-wide association study (GWAS) in 2006 identified a strong T2D susceptibility locus on chromosome 10; the strongest association marked by the microsatellite marker DG10S478 [3], and also by two common SNPs: rs7903146 and rs12255372, located in *TCF7L2* intron 3 and 4, respectively. Individuals homozygous for the risk-associated alleles were more than twice as likely to develop T2D as non-carriers. Subsequent studies have replicated this association in prospective and case–control cohorts in a range of populations [4–6].

**Figure 1:**
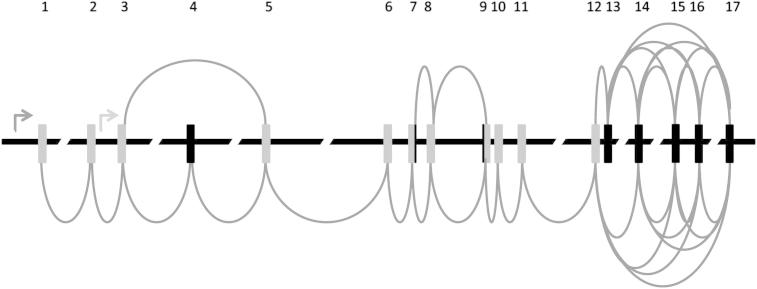
Structure of the *TCF7L2* gene. Arrows indicate transcription start sites. Grey bars indicate exons. Black bars indicate alternatively spliced exons. Round lines between exons indicate alternative splicing products.

TCF7L2 is a member of the high mobility group box family of transcription factors, activated by the WNT-signalling pathway. Illustrated in Fig. 1, the gene spans 215.9 kb and comprises 17 exons. The gene possesses two major domains: a catenin-binding domain (exon 1) and a central DNA-binding HMG domain (exons 10 and 11) [7]. At least five exons can be alternatively spliced [8] and most human tissues express detectable levels of this transcription factor [9]. Although the T2D-associated SNPs are located in non-coding regions it is not clear if these SNPs, or a variant in strong linkage disequilibrium (LD) with these, play a role in alternative splicing, gene expression, or protein structure.

The mechanisms leading to T2D from *TCF7L2* remain unknown, as indeed, to which cell types this ubiquitously expressed gene may be playing a role in disease pathogenesis. One obvious potential target tissue is the pancreatic islets, and a study of *TCF7L2* in this tissue demonstrated increased gene expression in risk allele carriers [10]. Another study investigated *TCF7L2* gene expression and splicing in additional potential target tissues: adipose and muscle tissue [11]. In contrast to the study in pancreatic islets, certain splice forms in subcutaneous adipose tissue were associated with reduced *TCF7L2* gene expression in individuals homozygous for the rs7903146 T risk alleles, but overall *TCF7L2* gene expression was not significantly associated with rs7903146 genotype.

It was relatively recently proposed that obesity, insulin resistance and T2D are chronic inflammatory diseases, and much research is currently involved with understanding the role of the body’s immune cells in metabolic imbalance [12]. The aim of this study was to determine whether the two T2D-associated SNPs were associated with alternative splicing or differences in gene expression levels, using peripheral blood mononuclear cells (PBMCs) as the potential target of *TCF7L2* expression in T2D pathogenesis. A previously reported pancreatic islet-specific functional polymorphism has been reported [13], however, this SNP is also associated with increased gene expression in a number of tissues, suggesting that either this SNP or another in strong LD has additional unreported functionality. We looked for further functional SNPs in LD with the *TCF7L2* SNPs associated with T2D from GWAS, that were not pancreatic-specific, since these may also have an as-yet unknown role in T2D pathogenesis. Locating all the causal variants will lead to a better capacity to predict disease, determine the mechanism of genetic susceptibility and guide the development of novel therapeutics.

## Methods

See supplement for a description of the methods used.

## Results

### Genotyping of rs7903146 and rs12255372

To investigate the association between *TCF7L2* genotype and mRNA splicing patterns a cohort of 100 healthy individuals were genotyped for the two SNPs associated with T2D risk (rs7903146 and rs12255372). The genotype distribution was in Hardy–Weinberg equilibrium and the frequencies consistent with previous studies [4–6] (rs7903146 (IVS3 Cgt; T): 55% CC, 37% CT, 8% TT, minor allele frequency (MAF) = 0.34, rs12255372 (IVS4 Ggt; T): 56% GG, 31% GT, 13% TT. MAF = 0.36).

### TCF7L2 exon 4 splicing

**Figure 2:**
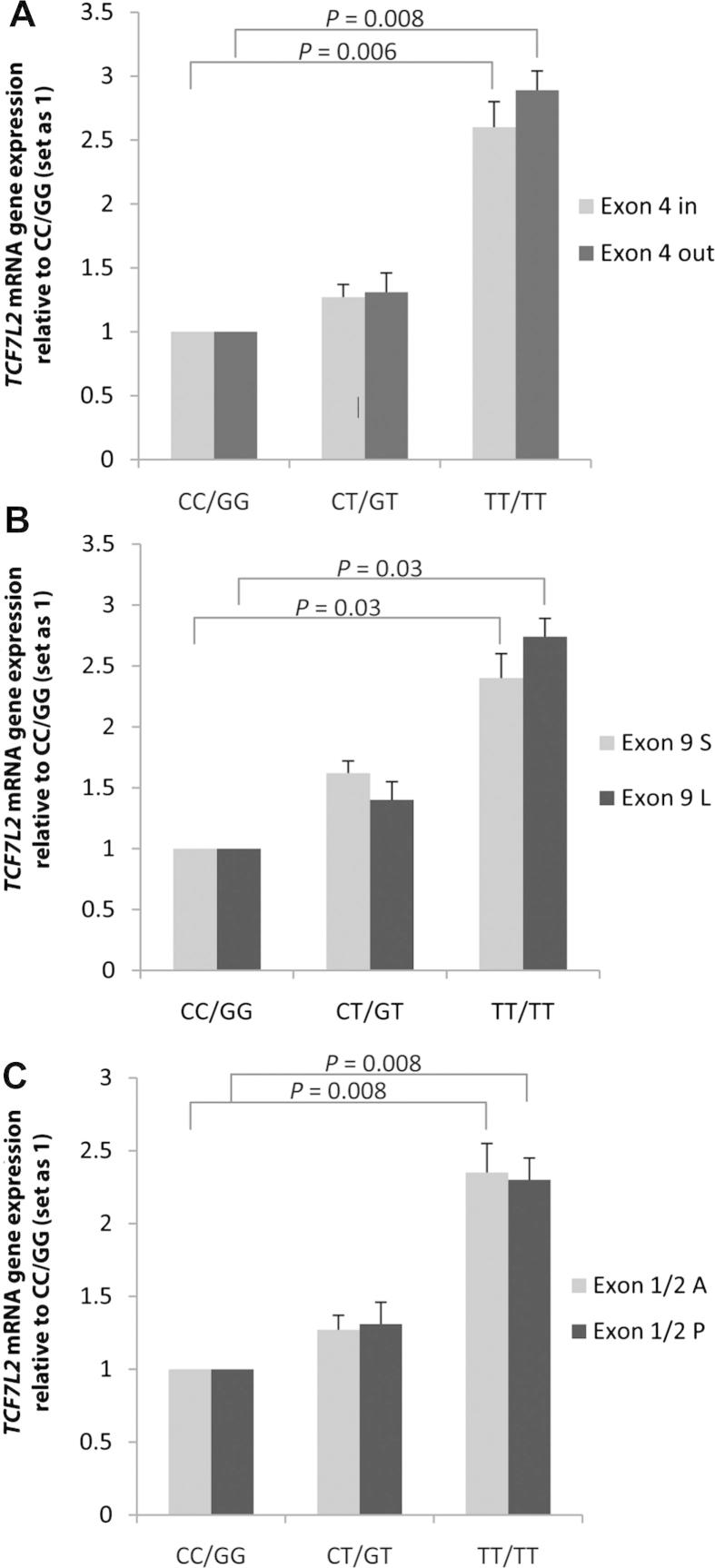
A, B and C. Relative expression (+S.E.M.) of *TCF7L2* mRNA transcripts by genotype, compared to the non-risk alleles CC (rs7903146) and GG (rs12255372). A) Comparison of *TCF7L2* transcripts with exon 4 present or exon 4 absent (*p*-value for trend using additive model: exon 4 in, *p* = 0.03; exon 4 out, *p* = 0.02) B) Comparison of *TCF7L2* transcripts with the exon 9 splice acceptor site present (exon 9 L) or absent (exon 9 S) (*p*-value for trend using additive model: exon 9S *p* = 0.01; exon 9 l, *p* = 0.04). C) Comparison of *TCF7L2* transcripts with exons 1 and 2 (exon 1/2 P) or without exons 1 and 2 (exon 1/2 A) (*p*-value for trend using additive model; exon 1/2A, *p* = 0.01; exon 1/2P, *p* = 0.008).

To examine splicing of *TCF7L2* exon 4 in individuals with *TCF7L2* T2D risk genotypes, a probe overlapping the exon 3/4 border and another overlapping the exon 3/5 border was used. The exon 3/4 probe identified PCR product from mRNA transcripts including exon 4, while the exon 3/5 probe identified mRNA transcripts that did not. No statistically significant difference between the relative proportions of exon 3/4 and exon 3/5 transcripts was observed when stratified by T2D risk genotypes (rs7903146 and rs12255372, Fig. 2A); indicating that alternative splicing of exon 4 was not affected by these T2D risk alleles. However, individuals homozygous for the rare alleles of both rs7903146 and rs12255372 expressed significantly more *TCF7L2* transcript than individuals homozygous for the common alleles (2.6-fold for transcripts with exon 4 *p* = 0 < 0.01 and 2.89-fold for transcripts lacking exon 4, *p* < 0.01).

### TCF7L2 exon 9 splicing

Exon 9 has an alternative splice acceptor site which adds 15 base pairs to its 5′ end. TaqMan probes were customized to span the exon 8/9 boundary, including or excluding the 15 base pairs. No statistically significant difference between rs7903146 and rs12255372 genotype and relative amounts of exon 9 short and exon 9 long was observed (Fig. 2B). Individuals homozygous for the rare allele expressed significantly more *TCF7L2* transcript than individuals homozygous for the common allele (2.4-fold for transcripts with the splice acceptor site *p* = 0.03 and 2.6-fold for transcripts lacking the additional 15 base pairs *p* = 0.032).

### Exon 1/2 splicing

To examine whether the putative splicing of exons 1 and 2 was associated with T2D risk alleles, two Taqman probes that spanned either the exon 1/2 boundary or the exon 5/6 boundary were used. The exon 1/2 probe spans exons 1 and 2 and the exon 5/6 probe was used to identify total mRNA transcript regardless of splicing, as exons 5 and 6 are not spliced. As shown in Fig. 2C, splicing of exons 1 and 2 does occur. However, as with exon 4 and 9, no statistically significant difference between rs7903146 and rs12255372 genotype and relative amounts of transcripts containing exons 1/2 and those without was observed. As with exon 4 and exon 9, homozygotes carrying the rare alleles express significantly more *TCF7L2* transcript than individuals homozygous for the common allele (2.25-fold increase in transcripts containing exons 1 and 2, 2.3-fold increase for transcripts without exons 1 and 2, *p* < 0.05 for both).

### Electrophoretic mobility shift assay

**Figure 3:**
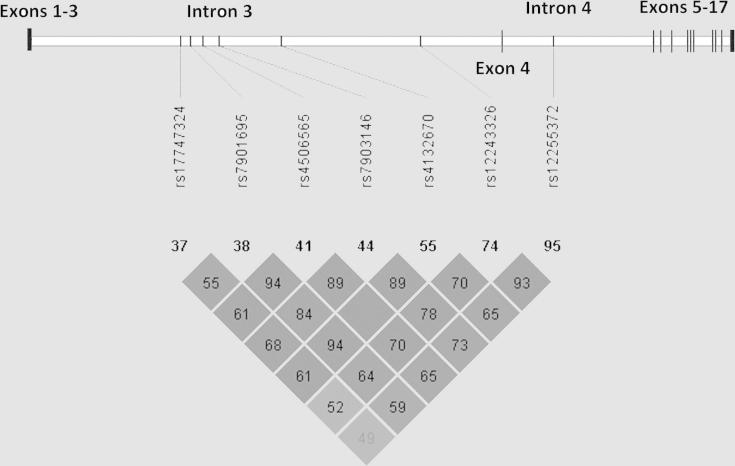
Linkage disequilibrium between SNPs in high LD with rs7903146 and rs12255372 and their relative positions in *TCF7L2* (Haploview).

**Figure 4:**
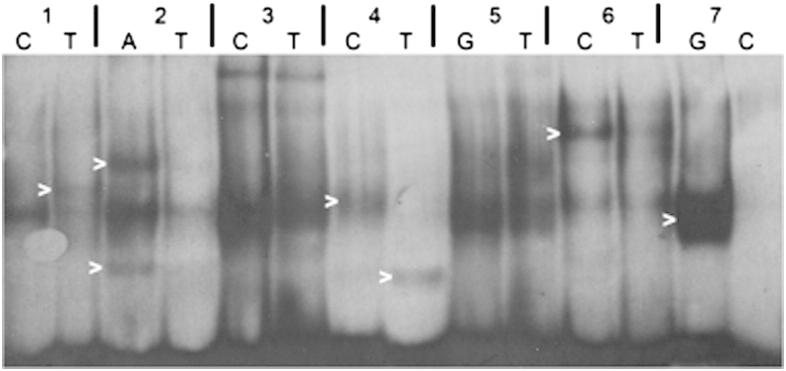
EMSA analysis to determine allele-specific binding differences. SNPs in strong LD with/including *TCF7L2* T2D risk SNPs. Allele-specific binding differences are highlighted. 1: rs4132670, 2: rs4506565, 3: rs12243326, 4: rs7903146, 5: rs12255372, 6: rs7901695, 7: rs17747324. Alleles are indicated above image.

Gene expression data suggested that *TCF7L2* T2D risk alleles act through gene expression levels rather than alternative splicing. To investigate whether differences in *TCF7L2* mRNA levels observed were due to differential transcription factor binding to *TCF7L2* risk alleles, electrophoretic mobility shift assays (EMSA) were performed. Linkage disequilibrium (LD) data from Caucasian populations (HapMap 3, release 2, http://gvs.gs.washington.edu/GVS/index.jsp and the 1000 Genomes Project [20]) were used to identify five additional SNPs in strong LD with the original GWAS SNPs associated with T2D (Fig. 3). EMSA was performed using probes that contained both alleles of all seven SNPs using nuclear extract the WiDr colon carcinoma cell line, a cell line that expresses high levels of TCF7L2. An allele-specific binding pattern was identified in five of these SNPs: rs4132670, rs4506565, rs7903146, rs7901695, rs17747324 (Fig. 4).

### Luciferase assay

Luciferase reporter assays examined whether variants that altered *in vitro* binding, also had allelic enhancer effects, influencing *TCF7L2* transcription. The original T2D-associated SNPs rs7903146 and rs12255372 were also included in this analysis. Allele-specific luciferase reporter constructs were designed with genomic sequences surrounding *TCF7L2* SNPs inserted downstream of the luciferase gene, driven by the *TCF7L2* minimal promoter. Luciferase activity was measured in the Huh7 and WiDr cell lines, two cell lines that express *TCF7L2*.

**Figure 5:**
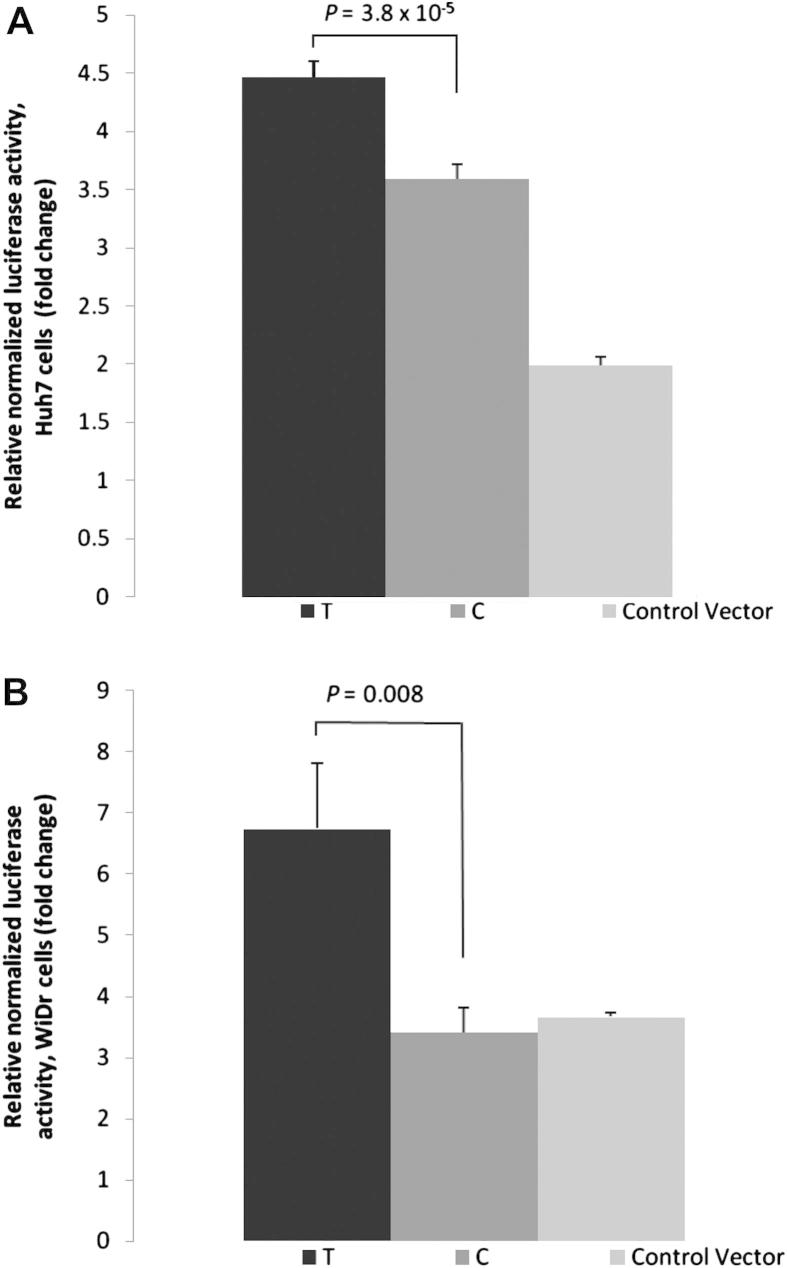
*TCF7L2* promoter expression (+S.E.M.) as a function of rs4132670 genotype. Expression levels are normalized to pGL3 basic vector with only the *TCF7L2* minimal promoter (control). A) Huh7 cells were transfected with pGL3 basic vectors containing the *TCF7L2* promoter with a fragment of rs4132670 subcloned into the enhancer site of pGL3. B) *WiDr* cells were transfected with pGL3 basic vectors containing the *TCF7L2* promoter with a fragment of rs4132670 subcloned into the enhancer site of pGL3.

When both cell lines were transfected with the T allele of the rs4132670 reporter constructs, significantly higher luciferase activity was observed compared to cells transfected with the C-allele (Huh7, 1.3-fold, *p* = 3.8 × 10^−5^; WiDr, 2-fold, *p* = 0.008) (Fig. 5A and Fig. 5B).

Of the remaining SNPs, there were no consistent allelic effects on expression in both cell types. For two SNPs, however, there were tissue-specific effects on enhancer activity. Huh7 cell lines transfected with the C-allele of rs4506565 displayed 1.3-fold lower luciferase activity when compared to cells containing the T allele (*p* = 5.6 × 10^−5^ Fig. S1a). Huh7 cell lines transfected with the T allele of rs12255372 displayed a 1.15-fold higher luciferase activity compared to cells transcfected with the G-allele (*p* = 0.02, Fig. S1b). There was no difference in luciferase activity for cells transfected with either of the rs7903146 alleles.

### In silico SNP analysis

For a functional SNP to act as an enhancer it is likely to occur in an accessible region of chromatin, such as that identified by DNase I hypersensitivity or formaldehyde-assisted identification of regulatory elements (FAIRE). Examination of annotated global maps of DNase I hypersensitivity in 22 cell lines [23] revealed that of the SNPs examined that altered DNA-binding, only rs4132670 was located within a DNase I hypersensitive region. The T allele of rs4132670 is in strong LD with the T allele of rs7903146. Resequencing this DNase I hypersensitive region in 7 healthy Caucasians of known genotype (3 CC, 2 CT, 2 TT individuals) did not identify any further variation that was in strong LD with the T2D risk SNP (data not shown).

### Multiplexed competitor EMSA

To determine the DNA-binding factor responsible for binding to the rs4132670 T allele, multiplexed competitor EMSA (MC-EMSA [14]) was carried out. This procedure screens for binding using cocktails of DNA competitors for a number of well-characterised DNA-binding proteins and included those predicted to bind through *in silico* software (MatInspector). MC-EMSA showed that rs4132670 T binding was competed out by the cis-inducible element (SIE) (Supplementary Fig. 4B and C).

## Discussion

A number of studies have replicated the findings by Grant et al. [3] demonstrating association between two intronic SNPs in *TCF7L2* and T2D [4–6]. The functionality of these SNPs and those in strong LD were explored using these markers with the aim of identifying further putative causal variants and the mechanism by which these may exert their effects.

Although *TCF7L2* transcription results in a number of alternatively spliced mRNA products, the causal effect of the T2D genetic risk variants are unlikely to be mediated by alternative splicing. There are no SNPs in high LD with T2D-associated SNPs located in splice-donor/acceptor sites, and there was no significant difference in splicing of exons 1/2, 4 or nucleotides 1–15 of exon 9 in PBMC mRNA between subjects of different genotype. The putative splice sites at exons 13–17 are in a region of very low LD from the T2D risk alleles, and are unlikely to be influenced by these SNPs [8]. This result is in line with previous studies which have found very little evidence for *TCF7L2* splicing to be associated with risk of T2D [15–17]. However, there was a significant association between the risk variants and increased *TCF7L2* gene expression levels. Individuals homozygous for the rare allele T at both SNP positions produce ∼2.5-fold the levels of *TCF7L2* transcript compared to wild-type individuals while individuals heterozygous for both alleles produced ∼1.5-fold more transcript. Our data derives from PBMCs, although it is consistent with findings from a similar study on pancreatic *TCF7L2* gene expression in risk allele carriers [10].

Interestingly, *TCF7L2* transcripts that do not contain exons 1 and 2 are produced in equal quantity as transcripts that do contain exon 1, regardless of genotype. Exon 1 codes for the β-catenin binding domain, and half the mRNA transcripts produced may therefore code for an isoform of *TCF7L2* that does not respond to β-catenin. It is unknown whether this variant is able to bind to *TCF7L2* target genes, and if so, whether it is able to participate in gene regulation.

A study by Mondal et al. [11] investigated *TCF7L2* gene expression and splicing in adipose and muscle tissue. The authors found that certain splice forms in subcutaneous adipose tissue are associated with reduced *TCF7L2* gene expression in individuals homozygous for the rs7903146 T risk alleles, but that overall *TCF7L2* gene expression was not significantly associated with rs7903146 genotype. This finding in adipose tissue is in contrast to our finding in blood, and may reflect tissue-specific differences in enhancer usage between adipose and blood tissues, and a SNP in strong LD with the rs7903146 that may affect alternative splicing only in adipose tissue. However, a previous study by Lyssenko et al. which measured *TCF7L2* mRNA in pancreatic islets from 7 T2D and 15 nondiabetic human cadaveric organ donors [10] found higher gene expression was associated with rs7903146 and rs12255372 T alleles. Individuals homozygous for the rare T alleles expressed 3.3-fold (*p* = 0.02) as much *TCF7L2* transcript as individuals possessing the common CC/GG genotype. The concordant results from this study in PBMCs and the study in pancreatic β-cells suggests that there may be a common mechanism by which the T2D risk alleles are influencing gene expression in these tissues.

Despite the relationship between the *TCF7L2* T2D risk alleles and increased gene expression, there is little evidence identifying causal allele/s. Gaulton et al examined the presence of putative regulatory elements specific to pancreatic islet cells [13]. Using FAIRE-seq, the authors identified ∼80,000 open chromatin sites of which ∼3300 were islet-selective. The *TCF7L2* SNP rs7903146 was located within an islet-selective open chromatin region. The T allele was found to show significantly greater enhancer activity than the C allele in two islet β-cell lines using reporter assays. However, this finding contradicts the allele-specific *TCF7L2* mRNA expression *common* to both pancreatic β-cells and PBMCs. We examined all SNPs in strong LD with rs7903146 or rs12255372 and performed EMSA and reporter assays on these. Cell lines used for these experiments were the colon and a liver cell line, which both expresses high levels of *TCF7L2* (data not shown), and were used to provide conformation of non-tissue-specific transcriptional effects. Of the three SNPs that influenced *TCF7L2* enhancer activity in the luciferase reporter assays, the rare C-allele of rs4506565 significantly decreased *TC7L2* transcription, contrary to the well-established observation that rs7903146 TT individuals produce more *TCF7L2* transcript. The rare T allele of rs12255372 was found to moderately (1.15-fold, *p* = 0.02) increase reporter expression levels in Huh7 cell only. In both cases, reporter assays in WiDr cell remained non-significant, indicating a possible liver tissue-specific response. The T allele of rs4132670 was associated with increased reporter expression levels in both WiDr and Huh7 cell lines.

EMSA analysis indicated that binding to rs4132670 T may be due to binding similarity to the cis-inducible element (SIE). SIE is known to bind STAT1-STAT1 and STAT1-STAT3 dimers [18]. The JAK-STAT pathway can be activated by IFN-γ and IL-6, along with several other cytokines, and there may be an interaction between these biomarkers and rs4132670: individuals that possess the rs4132670 T allele may have higher levels of STAT1-STAT3 binding to the *TCF7L2* enhancer, leading to increased expression levels, which will be exacerbated under chronic inflammatory stimuli, potentially leading to higher T2D in these individuals.

Although we looked at a relatively large number of PBMCs in our analysis of gene expression, we did not examine other tissues that may play a role in disease. However, the results from our *in vivo* expression assays using PBMCs were correlated with the expression levels of reporter gene in both liver and colon cells, indicating that this effect is unlikely to be tissue-specific. In addition to the functional analysis shown here, rs4132670 was located within a regulatory region identified by global mapping of DNase I hypersensitivity and FAIRE sites in a number of cell lines including aortic smooth muscle cells, liver, fibroblasts, lymphoblastoid cells, bronchial epithelial cells and epidermal keratinocytes [19] (supplementary Fig. 6). This gives additional lines of evidence to suggest that this area harbours an important regulatory region.

This candidate functional SNP is in strong LD with the two T2D risk SNPs identified by GWAS, with an r^2^ of 0.93 and 0.98 for rs7903146 and rs12255372, respectively, in Caucasians. The strong LD between rs4132670 and the T2D GWAS SNPs in Caucasians would mean that substitution by rs4132670 will, at best, result in only a modest improvement to T2D risk assessment, and to show such an effect would require a very large case–control study. Whether the two candidate functional SNPs rs7903146 and rs4132670 may act in concert or in an additive manner to affect disease risk remains to be determined.
