# Supplementary material for: Functional analysis of TCF7L2 genetic variants associated with type 2 diabetes
Source: Nutr Metab Cardiovasc Dis. 2013 Jun;23(6):550–6. doi: 10.1016/j.numecd.2011.12.012 (PMC3778915; doi:10.1016/j.numecd.2011.12.012)

Figure S1 a) Huh7 cells were transfected with pGL3 basic vectors containing the *TCF7L2* promoter with a fragment of rs4506565 subcloned into the enhancer site of pGL3.


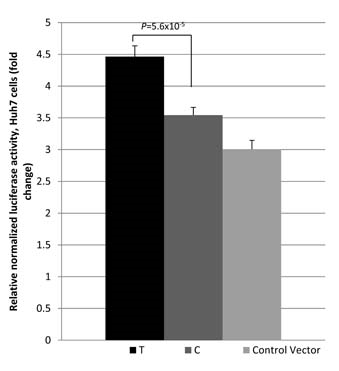


Figure S1 b) Huh7 cells were transfected with pGL3 basic vectors containing the *TCF7L2* promoter with a fragment of rs12255372 subcloned into the enhancer site of pGL3.


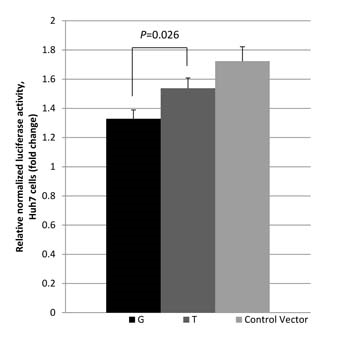

Supplement: Supplementary file 1 [file mmc1.doc]
