# Supplementary material for: Functional analysis of TCF7L2 genetic variants associated with type 2 diabetes
Source: Nutr Metab Cardiovasc Dis. 2013 Jun;23(6):550–6. doi: 10.1016/j.numecd.2011.12.012 (PMC3778915; doi:10.1016/j.numecd.2011.12.012)
Supplement: Supplementary file 2 [file mmc2.zip › numecd_959_mmc2.docx]

**Study Subjects**

100 healthy Caucasian volunteers were recruited from London, United Kingdom; all participants were healthy at the time of recruitment and consisted of 56 females and 44 males with a mean age of 32.3 years (±4.2 SD). All gave informed consent prior to inclusion to the study.

**Isolation of Peripheral blood mononuclear cells (PBMC), RNA and DNA extraction**

PBMCs were isolated from 10ml of blood using Lymphoprep (Axis-Shield). RNA was extracted using the RNeasy mini kit (Qiagen) according to manufacturer’s instructions. Total RNA was quantified using the NanoDrop ND-8000 full-spectrum UV/Vis spectrophotometer (NanoDrop Technologies). RNA quality was assessed using the Agilent 2100 Bioanalyzer (Agilent Technologies) and converted to cDNA using Superscript III Reverse Transcriptase according to manufacturer’s instructions (Invitrogen). Genomic DNA was extracted using the salting-out method (1) and quantified using the ND-8000 NanoDrop.

**Genotyping**

Genotyping of rs7903146 and rs12255372 was performed on genomic DNA using TaqMan Assay-by-Design SNP Genotyping Assays (Applied Biosystems). The allelic discrimination reactions were carried out on 384-well microplates using the Applied Biosystems 7900HT Real-Time PCR System with the TaqMan software.

**Gene Expression Quantification**

PBMC-derived *TCF7L2* mRNA levels and splicing patterns for each subject were examined using Taqman gene expression probes (Applied Biosystems). To examine the presence/absence of exon 4, probe A (Hs01009042_m1) spanned exons 3 and 4, probe B (HS01009053_m1) spanned exons 3 and 5. For examination of exon 9 alternative splicing, a custom gene expression probe (Probe C) and a made-to-order Probe D were used (Hs00181036_m1). Both probes spanned exons 8 and 9 but probe D included the 15 nucleotide alternative splice acceptor site at the start of exon 9. For the analysis of the putative alternative transcription start site in intron 1, two made-to-order gene expression Taqman probes were used spanning exon 1-2 (Hs01009040_g1) and exon 5-6 (Hs01009044_m1). Normalization of mRNA transcripts was carried out using three housekeeping genes (*GAPDH*, *ACTB* and *UBC*) and gene expression was analysed using the Rest Expression Software Tool by MW Pfaffl utilizing a Pair-Wise Fixed Reallocation Randomization Test (2).

**Tissue Culture**

WiDr colon carcinoma cells (European Cell Culture Collection) and Huh7 human hepatoma cells (JCRB Cell Bank) were cultured in Dulbecco's Modified Eagle Medium (PAA Laboratories) containing L-glutamine (200mM), non-essential amino acids (1x), penicillin-streptomycin (10.000 U/ml) and 10% foetal bovine serum, incubated at 37 C, 5% CO_2_.

**SNP selection for functional analysis**

All SNPs in strong LD (r^2^ > 0.8) with rs7903146 or rs12255372 were selected using the genome variation server (<http://gvs.gs.washington.edu/GVS>) based on HapMap 3 (release 2) data and 1000 Genomes Project pilot data (3).

***In silico* analysis**

SNPs were examined in *silico* for the presence of *in vivo* DNase I hypersensitive regions or formaldehyde-assisted isolation of regulatory elements (FAIRE) sites near these SNPs, using the ENCODE open chromatin data via the UCSC genome browser (http://genome.ucsc.edu, March 2006 assembly).

**Electrophoretic mobility shift assay (EMSA)**

Nuclear extracts from WiDr and Huh7 cell lines were obtained using the NE-PER Nuclear and Cytoplasmic Extraction Reagents kit (Pierce Biotechnology), according to manufacturer’s instructions, with the addition of Complete Protease Inhibitor (Thermo Scientific) to buffers CER I and NER I.  Probes were labelled using the Biotin 3’-End DNA Labelling Kit (Thermo Scientific) as described in the manual. Probe sequences are listed in supplementary data 2. Each EMSA binding reaction consisted of 2 μl 10x binding buffer (100mM Tris, 500 mM KCl; pH 7.5), 1 μg p[dI-dC], 0.5 μl 50mM MgCl_2_, 200 fmol biotin-labelled DNA, 5 μl nuclear cell extract made to a total of 20 μl with H_2_O, incubated at 25ºC for 30 min and followed by the addition of 5x loading buffer. Competition reactions were carried out with 30 min incubation on ice, prior to addition of labelled probes using 20nmol unlabelled competitor consensus sequences. Samples were loaded on to a 6% polyacrylamide gel and electrophoresed for 200 min at 150 mV using an Appleton Woods OmniPage VS20 EMSA gel tank. Transfer to positively charged Hybond-N^+^ nylon membrane (GE Healthcare) was achieved through Southern transfer (4) and detection was performed using the Chemiluminescent Nucleic Acid Detection Module (Pierce/Thermo Scientific).

**Luciferase reporter assay**

The minimal promoter region of *TCF7L2* (-800 to +512) was PCR-amplified and subcloned into the promoter site of a pGL3 basic vector (Promega) and verified by sequencing (see supplementary data 4 for primers). DNA Fragments ranging from 82bp to 109bp surrounding the SNPs (sequences available on request) were subcloned into the enhancer site of the pGL3 basic vector containing the minimal promoter region of *TCF7L2.* Transfections were carried out in Huh7 and WiDr cell lines in triplicate. Cells were co-transfected with a pRL-TK *Renilla* luciferase vector to control for transfection efficiency. Transfections were performed using Lipofectamine 2000 according to manufacturer’s recommendations. Cells were assayed 48 hours after transfection using the Dual-Luciferase Assay according to manufacturer’s instructions (Promega). A two-sided T-test was used to compare luciferase activity between alleles.

**Multiplexed-competitor EMSA**

Multiplexed-competitor EMSA was carried out as described previously (5). In brief, seven cocktails each consisting of 10 unlabeled DNA-based competitors to well-characterized DNA-binding proteins were added to each binding reaction prior to the addition of the labelled probe. Where a cocktail inhibited probe binding, the individual competitors of that cocktail were examined separately in a subsequent EMSA to identify the DNA-binding protein involved. Using the Genomatix ([www.genomatix.com](http://www.genomatix.com)) *in silico* predicted transcription factor binding changes of all SNPs, another 19 unlabeled DNA-based competitors were added in two further cocktails (sequences available on request).

References

1. Miller SA, Dykes DD, Polesky HF. A simple salting out procedure for extracting DNA from human nucleated cells. Nucleic Acids Res. 1988 Feb 11;16(3):1215.

2. Pfaffl MW, Horgan GW, Dempfle L. Relative expression software tool (REST) for group-wise comparison and statistical analysis of relative expression results in real-time PCR. Nucleic Acids Res. 2002 May 1;30(9):e36.

3. Durbin RM, Abecasis GR, Altshuler DL, Auton A, Brooks LD, Gibbs RA, et al. A map of human genome variation from population-scale sequencing. Nature. 2010 Oct 28;467(7319):1061-73.

4. Southern EM. Detection of specific sequences among DNA fragments separated by gel electrophoresis. J Mol Biol. 1975 Nov 5;98(3):503-17.

5. Smith AJ, Humphries SE. Characterization of DNA-binding proteins using multiplexed competitor EMSA. J Mol Biol. 2009 Jan 23;385(3):714-7.
