# Supplementary material for: Functional analysis of TCF7L2 genetic variants associated with type 2 diabetes
Source: Nutr Metab Cardiovasc Dis. 2013 Jun;23(6):550–6. doi: 10.1016/j.numecd.2011.12.012 (PMC3778915; doi:10.1016/j.numecd.2011.12.012)
Supplement: Supplementary file 3 [file mmc3.zip › numecd_959_mmc3.docx]

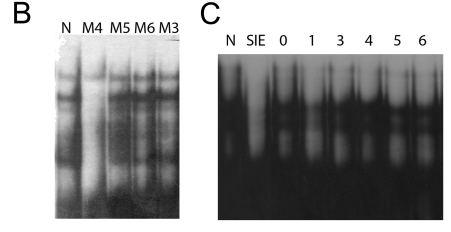


Supplementary Figure 4b. EMSA competition assay using multiplexed competitors for rs4132670 T. The first lane marked “N” has no competitors added. M4/5/6/3 contains multiplexed competitors (each containing 10 different consensus DNA sequences for well characterised DNA-binding proteins). The allele-specific band is eliminated with multiplex 4 DNA competitors.

Supplementary Figure 4c. EMSA competition assay with specific competitors from multiplex 4, competing with rs4132670 T. The first lane marked N has no competitors added. The only DNA sequence responsible for competition was from the SIE sequence.
