# Supplementary material for: Functional analysis of TCF7L2 genetic variants associated with type 2 diabetes
Source: Nutr Metab Cardiovasc Dis. 2013 Jun;23(6):550–6. doi: 10.1016/j.numecd.2011.12.012 (PMC3778915; doi:10.1016/j.numecd.2011.12.012)
Supplement: Supplementary file 4 [file mmc4.zip › numecd_959_mmc4.docx]

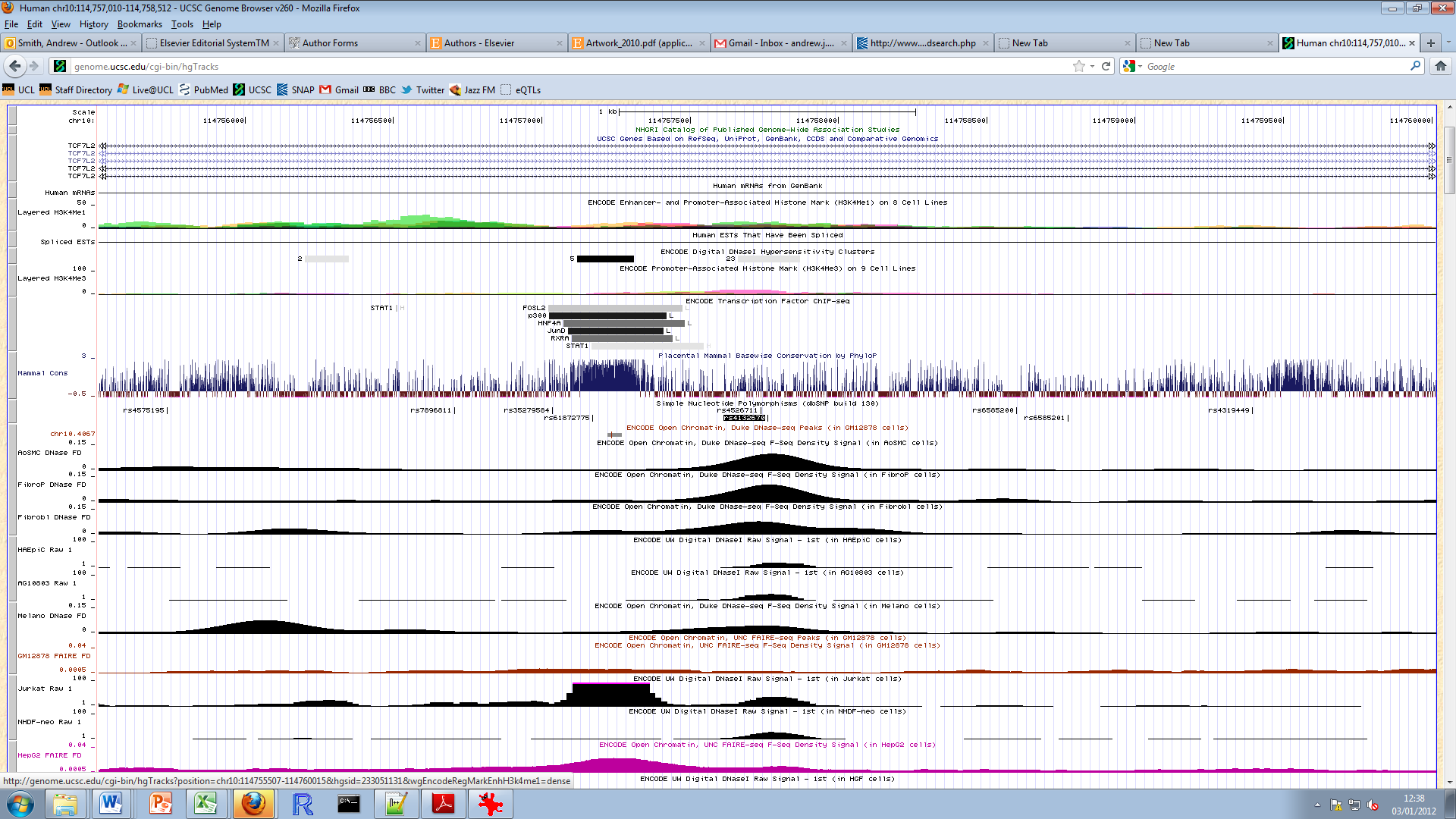


**Supplementary Figure 6.** Selected cell lines showing DNAse I hypersensitivity near rs4132670 in the ENCODE database. Dark bands indicate presence of DNase I hypersensitivity (http://genome.ucsc.edu/cgi-bin/hgGateway).
